# A curated soil fungal dataset to advance fungal ecology and conservation research in Australia and Antarctica

**DOI:** 10.1038/s41597-025-04598-5

**Published:** 2025-02-27

**Authors:** Luke Florence, Sean Tomlinson, Marc Freestone, John W. Morgan, Jennifer L. Wood, Camille Truong

**Affiliations:** 1 Department of Environment, Plant and Animal Science, La Trobe University, Bundoora, VIC 3083 Australia; 2 Biodiversity and Conservation Science, Department of Biodiversity, Conservation and Attractions, Kensington, WA 6151 Australia; 3 School of Biological Sciences, University of Adelaide, Adelaide, SA 5000 Australia; 4 The Biodiversity Consultancy, Cambridge, CB2 1SJ United Kingdom; 5 Department of Department of Microbiology, Anatomy, Physiology and Pharmacology, La Trobe University, Bundoora, VIC 3083 Australia; 6 Royal Botanic Gardens Victoria, Melbourne, VIC 3004 Australia

**Keywords:** Ecological modelling, Biodiversity, Microbial ecology, Biogeography

## Abstract

DNA metabarcoding has played a pivotal role in advancing our understanding of the diversity and function of soil-inhabiting fungi. The Australian Microbiome Initiative has produced an extensive soil fungal metabarcoding dataset of more than 2000 plots across a breadth of ecosystems in Australia and Antarctica. Sequence data requires rigorous approaches for the integration of species occurrences into biodiversity platforms, addressing biases due to false positives or overinflated diversity estimates, among others. To tackle such biases, we conducted a rigorous analysis of the fungal dataset following best practices in fungal metabarcoding and integrated it with over 100 predictor variables to fast-track data exploration. We carefully validated our methodology based on studies conducted on historical versions of the dataset. Our approach generated robust information on Australian soil fungi that can be leveraged by end-users interested in biodiversity, biogeography, and conservation. This novel resource will unlock new frontiers in soil fungal research within the Southern Hemisphere and beyond.

## Background & Summary

Soil-inhabiting fungi play an indispensable role in shaping the composition and function of terrestrial ecosystems^1^. As dominant drivers of soil carbon and nutrient cycles, fungi sustain plant production through a stable supply of available nutrients^2^. Mycorrhizal fungi influence plant diversity^3^, distribution^4^, and productivity^5,6^ by facilitating nutrient uptake, improving pathogen resistance, and promoting overall ecosystem health^7^. While soil-borne fungal pathogens can pose threats to global food security and ecosystem resilience in the face of global environmental change^8,9^, they can also promote biodiversity^10,11^ by engaging in antagonistic interactions with plants and animals. To address environmental challenges, it is essential to harness our understanding of soil fungal biodiversity, as the balance between functional guilds is vital to ensure ecosystem stability^12^, improve restoration outcomes^13^, and support sustainable agriculture initiatives^14^. However, achieving robust research to inform environmental policy and management requires the establishment of comprehensive protocols and well-curated information that can accurately capture fungal diversity^15^.

Fungi represent a megadiverse kingdom dominated by inconspicuous taxa that remain largely undetected by the naked eye^16^. Traditionally, observational approaches to documenting fungi have limited our attention to groups that produce visible reproductive structures, such as mushrooms, or fungi that can be isolated and grown under laboratory conditions^17,18^. The advent of high-throughput DNA sequencing methods, including metabarcoding, has dramatically improved the detection and understanding of fungal diversity in a variety of ecosystems worldwide^19,20^. The capacity to determine the species composition of fungi within a given environmental sample has revealed, for example, new fungal phyla and their hidden ecological functions^16,17^.

**Fig. 1:**
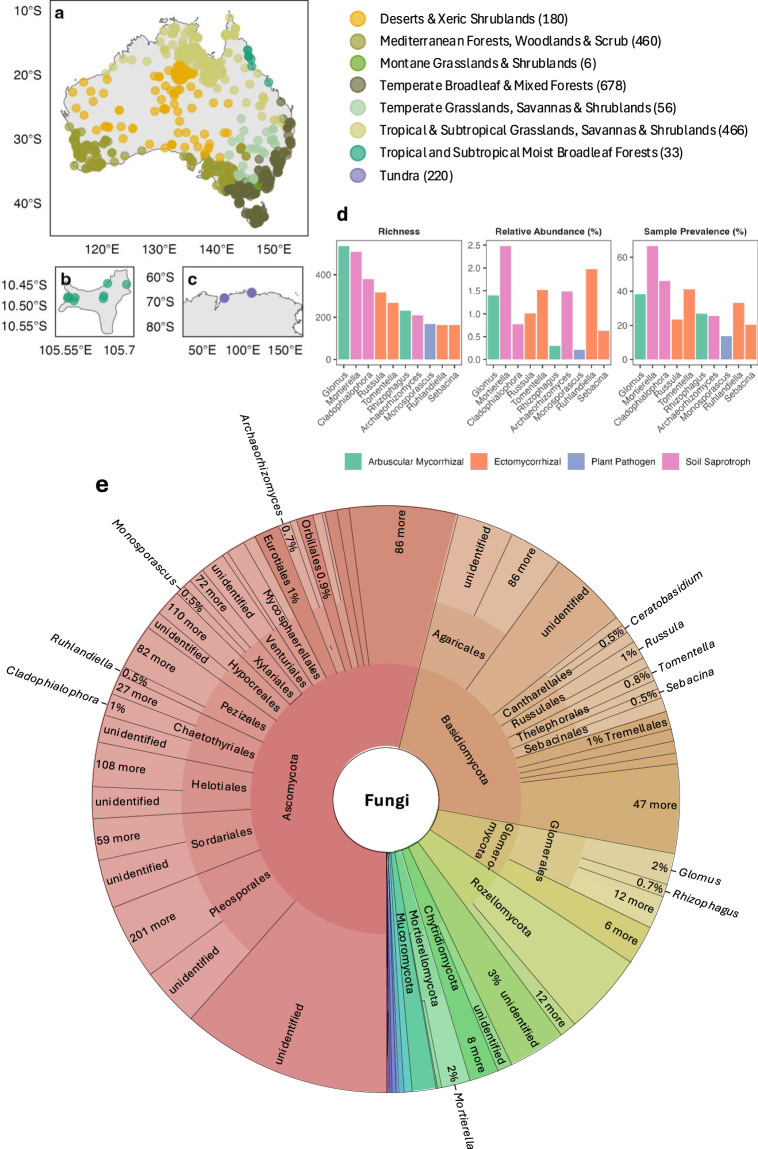
Geographic and taxonomic distribution of ITS1 fungal operational taxonomic units (OTUs) from our contemporary Australian Microbiome dataset. Plot locations (*n* = 2,103) of Australian Microbiome samples collected from terrestrial biomes in eight ecoregions, with the number of samples per ecoregion in parenthesis: (**a**) continental Australia (*n* = 1,874), (**b**) Christmas Island (*n* = 8) and (**c**) Antarctica (*n* = 220). (**d**) OTU richness, sequence abundance, and sample prevalence of the top ten most OTU-rich fungal genera colour-coded by their primary guilds. (**e**) KRONA chart showing the taxonomic distribution of dominant fungal phyla, orders, and species; an interactive chart for all taxonomic groups and ranks is provided on figshare^57^.

The Australian Microbiome Initiative aims to promote microbiome research by developing publicly available metabarcoding data focused on four main groups of organisms: bacteria, archaea, eukaryotic microbes, and fungi^21^ (www.australianmicrobiome.com) This initiative has primarily focused on sampling terrestrial topsoil (0–10 cm) and subsoil (20–30 cm), as well as marine samples from coastal and pelagic zones. Using Illumina amplicon paired-end sequencing, the Australian Microbiome has generated fungal metabarcodes targeting the internal transcribed spacer region (ITS), the genetic marker of choice for fungal metabarcoding^22^. ITS amplicons generated from topsoil samples cover 2,225 uniquely georeferenced sites so far (Fig. 1), spanning an extraordinary variety of bioregions, vegetation classes, and land use types. This dataset has been used to address a range of fundamental and applied research questions, including unravelling new fungal records in Australia^23^, evaluating diversity patterns in soils^24^, modelling the distributions of fungal species^25^, monitoring fungi for revegetation applications^26,27^ and human health purposes^28^, as well as exploring correlations between fungal diversity and community assembly with a range of environmental predictors^29–33^, disturbance scenarios^34–36^ and vegetation types^37^.

Sequence artefacts (or false positives) can occur at various stages of a metabarcoding project, from sample collection to bioinformatic analysis. These artefacts include biological contamination, chimera formation during library preparation, incorrect base calls (or sequencing errors) during sequencing, misassignment of sequences to samples (or index switching), or taxonomic misidentifications^38^. Due to sequence length limitations of Illumina platforms, most fungal metabarcoding studies, including large-scale and global datasets^39–41^, typically target the ITS1 or ITS2 subregions independently. Merging complementary forward and reverse sequences is a critical sequence quality filter to reduce sequencing errors, thereby improving the accuracy of diversity estimates^38,42^. The chosen polymerase chain reaction (PCR) primers of the Australian Microbiome, ITS1F^43^ and ITS4^44^, target the full ITS region, leading to two amplicons (i.e. ITS1 for forward sequences and ITS2 for reverse sequences) from which sequences were generally too short to be merged^45^. Consequently, the GlobalFungi^46^ database—a comprehensive atlas of global fungal distribution comprised of hundreds of individual metabarcoding studies—revealed extremely high levels of richness in well-described fungal genera known for their relatively low diversity levels^47^. Yet this inflated diversity was mostly attributed to single-end sequences from the Australian Microbiome ITS dataset^47^, underscoring their uneven quality when processed using routine bioinformatic techniques. When analysing the bacterial component of the Australian Microbiome dataset, richness estimates in individual samples were highly dependent upon the overall diversity of the set of samples sequenced together (i.e. within a sequencing library), a phenomenon mostly attributed to index switching^48^. Processed Australian Microbiome ITS data were integrated into the Atlas of Living Australia^49^ (ALA), which is transferred to the Global Biodiversity Information Facility (GBIF). Consequently, misleading occurrences of exotic *Amanita* species in Australia were detected in the ALA and GBIF, which were attributed to lenient settings during the taxonomic assignment of Australian Microbiome ITS data^50^. Furthermore, ALA and GBIF now house more than 1,000 material sample records of ectomycorrhizal fungi in Antarctica sourced from the Australian Microbiome ITS dataset^51,52^, despite the absence of ectomycorrhizal host plants^53^, human observations, or specimen records to corroborate these findings^54^. Together, these studies highlighted the need to re-analyse the Australian Microbiome ITS dataset for their integration into biodiversity platforms, to achieve accurate fungal detections and robust ecological conclusions.

**Fig. 2:**
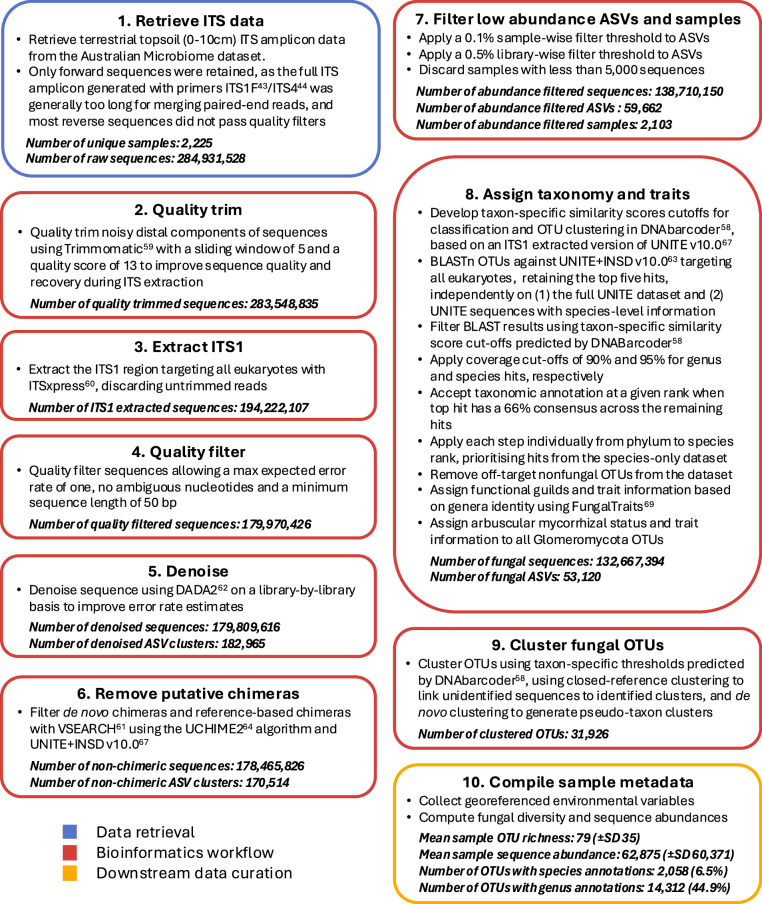
Study workflow: Data retrieval, bioinformatics analysis, and compilation of sample metadata and environmental predictors for generating our contemporary Australian Microbiome ITS1 dataset.

To provide novel and robust insights into fungal biodiversity in Australian and Antarctic soils, we meticulously reanalysed all ITS topsoil (0–10 cm) samples currently available from the Australian Microbiome, following the most up-to-date protocols and recommendations for fungal metabarcoding^38,42,55,56^ (Fig. 2). We established a detailed and reproducible bioinformatic pipeline and carefully benchmarked our results by evaluating the impact of data processing on fungal diversity, ectomycorrhizal occurrences, and *Amanita* taxonomy, based on previous studies^47,48,50^. We further validated our dataset by reproducing a study that modelled fungal species distributions based on a historical version of the Australian Microbiome dataset^25^. With the aim of boosting research on the diversity, ecology, and conservation of soil fungi in Australia, we provide a reliable dataset that is readily transferable to end-users interested in exploring, modelling, or conserving fungi in soils. Our conservative approach is best suited for the integration of sequence data into biodiversity platforms such as ALA or GBIF and will benefit future research on soil fungi in Australia and beyond.

## Methods

We provide a summary of our workflow in Fig. 2. All reproducible scripts are available on GitHub (see Code Availability), and primary data files and OTU matrices are on a dedicated data repository^57^.

### Data collection

We retrieved ITS amplicon data from topsoil (0–10 cm layer) samples generated from terrestrial soil biomes by the Australian Microbiome Initative^21^ (www.australianmicrobiome.com) and available on the Bioplatform Australia data portal (https://data.bioplatforms.com/organization/australian-microbiome) in January 2024, using search terms “sample_type:Soil & amplicon:ITS & depth_lower:0.1”. Accession numbers and persistent URL links for each sample are available in the sample metadata file^57^. The original data we sourced are openly available for re-use and re-distribution under Creative Common Attribution License: see the Australian Microbiome data sharing policy (https://www.australianmicrobiome.com/protocols/data-policy/) and references in Table 1 for the georeferenced grid data. Detailed soil sampling and sequencing protocols with historical updates are available on the Australian Microbiome website (https://www.australianmicrobiome.com/protocols).

**Table 1:** Summarised list of sample metadata from our contemporary Australian Microbiome dataset.

Dataset	Category	Variable description
This study	Fungal diversity and abundance	Observed richness, Shannon diversity and absolute read abundance
This study	Proxy to mould contamination	Relative abundance of Mortierellales, Umbelopsidales and *Trichoderma*
Australian Microbiome Initiative	Source file	Download links to original sequence files and sample metadata
Bioclimatic variable suite for continental Australia^76^	Climate	35 climatic variables including temperature, precipitation, radiation and moisture index
Global maps of soil temperature^77^	Soil climate	11 soil temperature variables at two soil depths (0–5 and 5–15 cm)
Australian Microbiome	Soil chemistry	13 measured soil variables including pH, conductivity, carbon and mineral nutrients
Soil and landscape grid national soil attribute maps^82–87^	Soil chemistry	14 soil variables including pH, carbon fractions and nutrients at two soil depths (0–5 and 5–15 cm)
Australian Microbiome	Soil physical	Measured soil variables for texture, sand, silt, and clay
Soil and landscape grid national soil attribute maps^78–80^	Soil physical	5 soil variables including water capacity, density and textures at two soil depths (0–5 and 5–15 cm)
Soil and landscape grid national soil attribute maps^81^	Soil physical	Soil depth
Australian Microbiome	Plant communities	Vegetation types
Vegetation height and structure^88^	Plant communities	3 vegetation structure variables for vegetation classification, cover and height
Plant diversity spatial layers for Australia^89^	Plant communities	3 variables for plant species richness, compositional dissimilarity and diversity importance
Estimation of habitat condition for terrestrial biodiversity^90^	Habitat condition	3 variables for habitat condition, local pressure and ecosystem condition
Australian Microbiome	Geography	Elevation
Digital elevation model^91^	Geography	Elevation
Australian Microbiome	Geography	Absolute longitude and latitude
Australian Microbiome	Temporal	Sample collection date

A comprehensive list is detailed in the sample metadata descriptor file on figshare^57^.

### Bioinformatics workflow

This workflow is informed by up-to-date recommendations for fungal metabarcoding^38,42,55,58^. We retained demultiplexed sequences from 2,443 samples representing 2,225 uniquely georeferenced plots from 42 sequencing libraries. We initially trimmed sequences using Trimmomatic^59^ v0.36 to eliminate noisy distal components of sequences, thereby improving sequence quality scores and the recovery of ITS1 sequences during ITS extraction. The full ITS1 subregion was extracted using ITSxpress^60^ v2.0.0 (all eukaryotes) and a minimum sequence length > 50 bases; partial ITS1 sequences were discarded as too noisy (see Technical Validation: Impact of ITS1 length on detection). ITS1 sequences were quality filtered using a maximum expected error rate of one, allowing zero ambiguous nucleotides in VSEARCH^61^ v2.22.1. Denoising was performed library-by-library to improve error rate estimates using DADA2^62^ v1.30. We removed putative chimeras using the denovo method and reference-based method against UNITE + INSD^63^ v10.0 (all eukaryotes) using the UCHIME2^64^ algorithm in VSEARCH^61^.

We acknowledge that we were unable to perform some recommended quality filtering steps due to compromised sequence data. For example, we could not merge complimentary forward and reverse sequences because the combined length of forward and reverse sequences was typically shorter than the full ITS (ITS1–5.8S–ITS2) amplicons that were targeted with the chosen PCR primers (ITS1F^43^ and ITS4^44^). Since the single-end forward sequences (i.e. ITS1 sequences) were superior in quality to the single-end reverse sequences (i.e. ITS2 sequences), we relied on single-end forward sequences that captured the ITS1 region, as did most previous studies using Australian Microbiome data^23–37^. Assessing index switching rates based on positive control samples is another important filtering step^38,56^, as index switches represent one of the most detrimental artefacts in the evaluation of biogeographical patterns^65^. Because positive control samples only occurred in 24 of the 42 sequencing libraries generated by the Australian Microbiome, this limited our ability to rigorously detect index switches. To adapt to these methodological constraints, we chose a conservative approach when processing the sequence data to minimise the likelihood of retaining false positives. This conservative approach included denoising using the DADA2^62^ algorithm, a haplotype-based approach (i.e. amplicon sequence variant or ASV) developed to cluster ribosomal RNA gene amplicons based on estimated rates of sequencing errors^55,62^. To achieve robust inferences, we quality filtered sequences using a maximum expected error rate of one, as the default settings of DADA2^62^ (max error = 2) led to inflated diversity and unreliable species distributions. While denoising significantly reduces the proportion of sequence artifacts^62^, it can also underestimate the richness of rare and phylogenetically unique fungi by incorrectly identifying low abundant ASVs as noise^38,42^. This discrimination is likely to disproportionately eliminate early diverging fungi, while inflating the proportional richness of dominant fungal groups within the phyla Ascomycota and Basidiomycota^38^. We found this to be the case in our analysis (see Technical Validation: Impact of abundance filtering approaches) and recognise that our conservative approach will result in some biases in fungal diversity estimates.

### Curation of the sample-by-ASV matrix

To curate the sample-by-ASV matrix, we applied two sequential filters: (1) A sample-wise abundance filter removed ASVs with relative abundance < 0.1% of the total sequence count within each sample to address sequencing errors as well as environmental and wet lab contaminations^56^. (2) A library-wise abundance filter removed ASVs from individual samples where thier abundance was < 0.5% of the total ASV’s sequence count across the entire library. This library-wise filter targeted index-switching artifacts, which manifest as low-abundance ‘bleed-through’ of ASVs into non-source samples^56^. We established these thresholds (1) based-on positive control richness, (2) by assessing ectomycorrhizal distributions in Antarctic samples where ectomycorrhizal host plants are known to be absent, and (3) by eliminating putative artefacts while minimising relative taxonomic biases between abundant and rare taxa (see Technical Validation: Impact of abundance filtering approaches). We further eliminated positive controls on a library-by-library basis. Finally, we removed plot replicates by retaining the sample with the lowest richness-to-abundance ratio. We also removed low-abundance samples with sequencing depth < 5000 sequences, since low-abundance samples tend to accumulate greater proportions of sequence artefacts than high abundance samples^38^. We acknowledge that our abundance filtering approach is fairly stringent and can result in false negatives and an overall underestimation of fungal diversity (see Technical Validation: Impact of abundance filtering approaches). However, we sometimes detected extremely high index switching rates in the sequence data prior to filtering, based on mock community samples with a mean richness of 141 (±123), instead of an expected OTU richness of 10. We therefore focused on limiting false positives that are more deleterious when informing biodiversity platforms, such as ALA or GBIF^66^, or for ecological inferences such as niche and distribution modelling^65^.

### Taxonomic and trait assignments

**Fig. 3:**
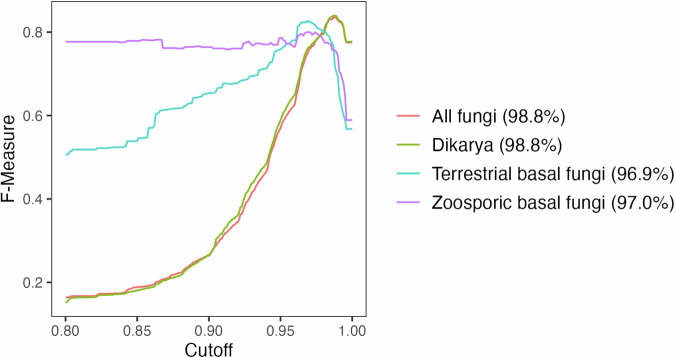
Species-level global similarity scores as predicted by DNAbarcoder^58^ using unique fungal sequences in UNITE + INSD^67^ v10.0. The analysis covered three major fungal groups: Dikarya (phyla Ascomycota, Basidiomycota and Entorrhizomycota); terrestrial early diverging fungi (phyla Basidiobolomycota, Calcarisporiellomycota, Entomophthoromycota, Glomeromycota, Kickxellomycota, Mortierellomycota, Mucoromycota and Zoopagomycota); and single-celled zoosporic fungi (phyla Aphelidiomycota, Blastocladiomycota, Chytridiomycota, Monoblepharomycota, Neocallimastigomycota, Olpidiomycota, Rozellomycota and Sanchytriomycota, as well as GS01). Global similarity scores for each group are shown in parentheses. These results demonstrate that kingdom-level fungal scores are skewed toward Dikarya, and more accurate global scores of early diverging fungi can be achieved by analysing these groups separately.

We used DNAbarcoder^58^ to predict taxon-specific similarity score cut-offs for taxonomic assignments from rank phylum to species, using ITS1-extracted fungal sequences in UNITE + INSD^67^ v10.0. Local cut-offs were set for each supertaxon–subrank combination (e.g. class cut-off for subrank class in supertaxon Ascomycota) where at least ten taxa were represented by 30 sequences, and the max proportion of sequences attributed to any individual taxon was less than 50%. Local cut-off predictions for supertaxon–subrank combinations were computed across all higher ranks (e.g. species-level cut-offs were predicted for *Cotinarius*, Cortinariaceae, Agaricales, Agaricomycetes and Basidiomycota). Final cut-offs were retained for each supertaxon–subrank combination, giving preference to cut-offs with the highest confidence. When local cut-offs could not be obtained due to insufficient number of sequences or taxa within a subgroup, a global cut-off was predicted. Because the global predictions were highly biased toward Dikarya, we subdivided the dataset based on phylogenetic relatedness to improve global cut-off predictions from class to species (Fig. 3). Phylum-level cut-offs were predicted for all fungi, and kingdom-level cut-offs were derived from these phylum cut-offs.

We assigned taxonomy to OTUs by initially running BLASTn queries (-task blastn-short in BLAST+^68^ v2.14.1) against an ITS1 extracted version of UNITE + INSD^63^ v10.0 and retaining the top five hits. This process was independently applied to the full UNITE + INSD dataset, as well as a filtered version containing sequence species-level information. We used the resulting taxonomy tables to assign taxonomy from kingdom to species using a three-step approach. First, we computed similarity scores using BLAST percent identify as outlined in DNAbarcoder^58^. This approach adjusts for short alignment lengths to mitigate artificially inflated high similarity scores that result from such alignments. Second, we filtered BLAST hits using taxon-specific cut-offs predicted by DNAbarcoder^58^, and coverage cut-offs of 90% for genus and 95% for species to avoid misassignments based on high similarity scores and partial alignments. Third, taxonomy was accepted at a given rank if the remaining BLAST hits achieved over 66% consensus at that rank, with preference given to hits from the species-only UNITE + INSD dataset. The resulting taxonomy table was annotated with trait information based on generic identity in FungalTraits^69^, except for arbuscular mycorrhizal traits which were assigned to all Glomeromycota ASVs^70^.

We clustered ASVs into OTUs to account for intraspecific and intragenomic sequence variants in the ITS region^38,42,55,71^, using a taxonomically-informed approach adapted from previous research^72–74^. This approach clustered sequences from ranks kingdom to species using the taxon-specific cut-offs we predicted with DNAbarcoder^58^. Initially, closed-reference clustering was performed with ASVs having taxonomic affiliations serving as cluster cores, while unidentified ASVs were matched to their nearest cluster core using BLASTn (-task blastn-short in BLAST+^68^ v2.14.1) and the taxon-specific sequence similarity cut-offs. Similarity scores were adjusted for short alignment lengths following the methods outlined in DNAbarcoder^58^. This step iterated until no new matches were found, forming approximate single-linkage clusters. Single-linkage *de novo* clustering was then performed on the remaining unclustered ASVs using BLASTClust^75^ v2.2.26 and each *de novo* cluster was tagged with unique pseudotaxon names (e.g. ‘pseudo_class_Ascomycota_1234’ for an unidentified class cluster with taxonomic affiliation to Ascomycota). Reference-based and *de novo* clustering required 90% and 95% sequence coverage at genus and species ranks, respectively. This process was performed in a nested fashion from kingdom to species, with each step constrained by a given supertaxon, and species-level clusters represented OTUs. The final dataset^57^ contained 31,926 fungal OTUs and 2,104 unique samples^58^.

### Collation of environmental predictors

To facilitate data exploration by end-users, we supplemented sample metadata with more than 120 predictor variables from a variety of data sources (Table 1). These predictors include sample- soil physiochemical measurements from the Australian Microbiome, as well as georeferenced predictors representing climate^76^, soil temperature^77^, soil physiochemistry^78–87^, vegetation structure^88^, plant diversity^89^, habitat condition^90^, and geographic variables^91^. We also provided references to source raster files of georeferenced variables to assist predictive modelling applications.

## Data Records

All primary data products produced in our study are available at figshare^57^ in eight files: (1) sample metadata file, (2) sample metadata descriptor, (3) sample-by-OTU matrix with absolute sequence abundances, (4) sample-by-OTU matrix normalised to a minimum sequencing depth of 5000 reads, (5) quality-filtered ITS1 reference sequences of OTUs in FASTA format, (6) taxonomy file without pseudotaxon names (i.e. pseudotaxa renamed as ‘unidentified’), (7) taxonomy file with pseudotaxon names, and (8) interactive KRONA chart showing the taxonomic distribution of our contemporary Australian Microbiome dataset. The sample metadata contains persistent links to the source files in fastq format, information on sample location, date of collection, a range of measured soil physicochemical characteristics, georeferenced environmental variables, alpha-diversity statistics, and estimated level of mould contamination (Table 1). The taxonomy file contains information on representative BLAST hits for each OTU, along with the associated trait information and relevant taxonomic assignment statistics (Table 2).

**Table 2:** Summarised information from the taxonomy file of our contemporary Australian Microbiome dataset.

Variable type	Variable description
Taxonomic information	UNITE + INSD^67^ phylum to species annotations and the associated reference sequence accession code
OTU abundance	Absolute OTU sequence abundance
Assignment information	DNABarcoder^58^ predicted cut-off used for taxonomic assignment and the lowest rank with taxonomic annotation
Trait information	16 variables covering ecological, morphological, functional, and evolutionary traits based on FungalTraits^69^

A comprehensive list is detailed in the information file on figshare^57^.

## Technical Validation

### Data processing

#### Impact of ITS1 length on detection across taxonomic groups

**Fig. 4:**
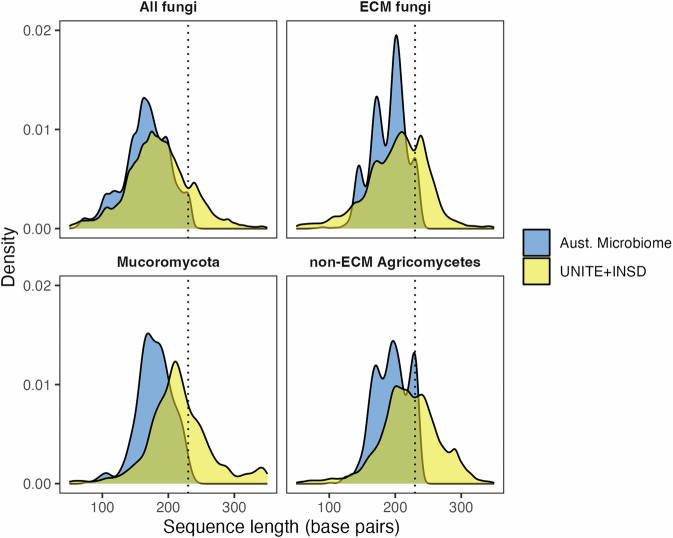
Distribution of unique ITS1 fungal sequence lengths across UNITE + INSD^67^ v10.0 and in our contemporary Australian Microbiome dataset, comparing all fungi, phylum Mucoromycota, ectomycorrhizal (ECM) fungi, and non-ECM (primarily saprotrophic) Agaricomycetes. In these groups, we expect that the exclusion of partial ITS1 sequences during ITS extraction can reduce species detection and biase diversity estimates. The dotted line at 230 bp indicates the 99th percentile of ITS1 sequence length in our contemporary Australian Microbiome dataset, suggesting that species with longer sequences are likely to remain undetected.

**Fig. 5:**
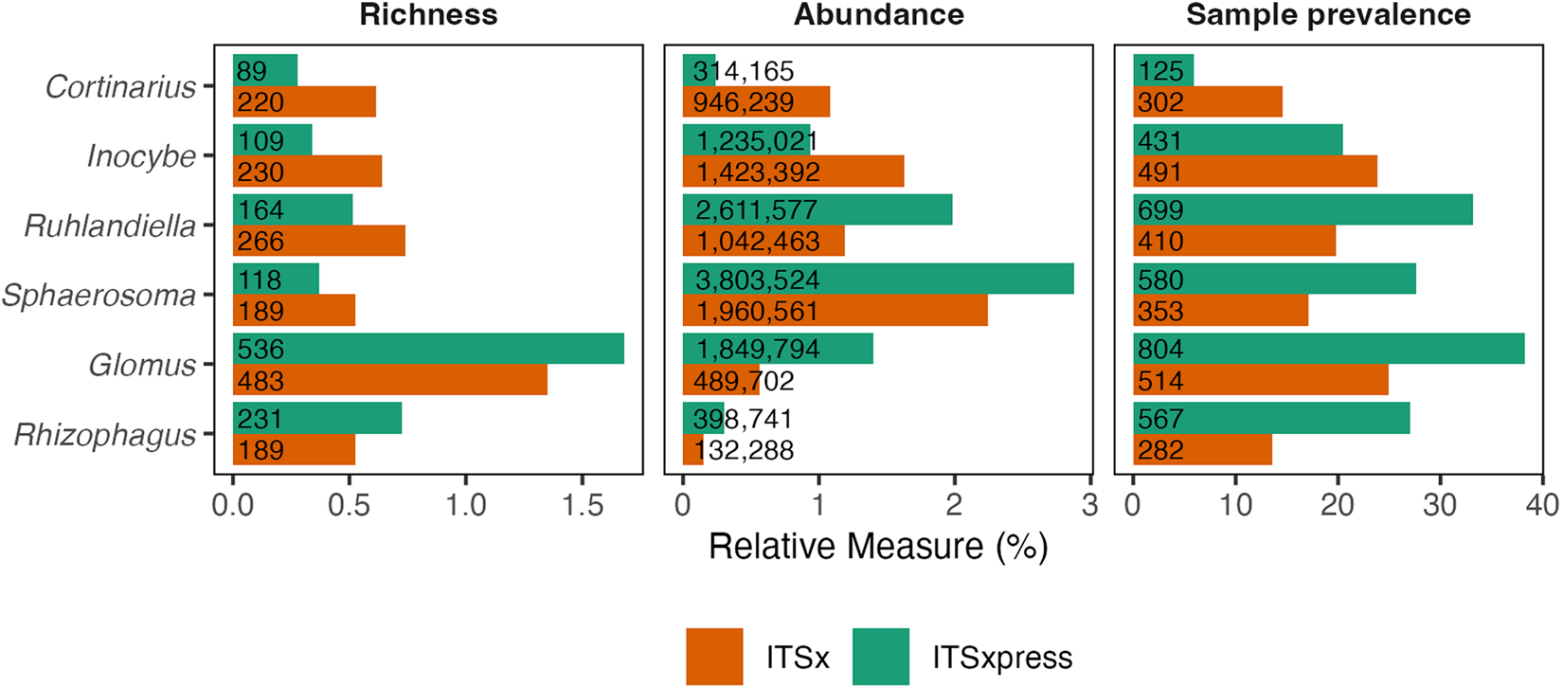
Impact of ITS1 extraction methods on operational taxonomic unit (OTU) richness, sequence abundance, and OTU prevalence in our contemporary Australian Microbiome dataset. The ITSx^95^ method includes full and partial ITS1 sequences, whilst ITSxpress^60^ retains only full ITS1 sequences. We selected ITSxpress as the preferred approach due to reduced noise in the final dataset compared to ITSx. The x-axis displays relative values across the global dataset, with numbers in bars showing absolute measures.

**Fig. 6:**
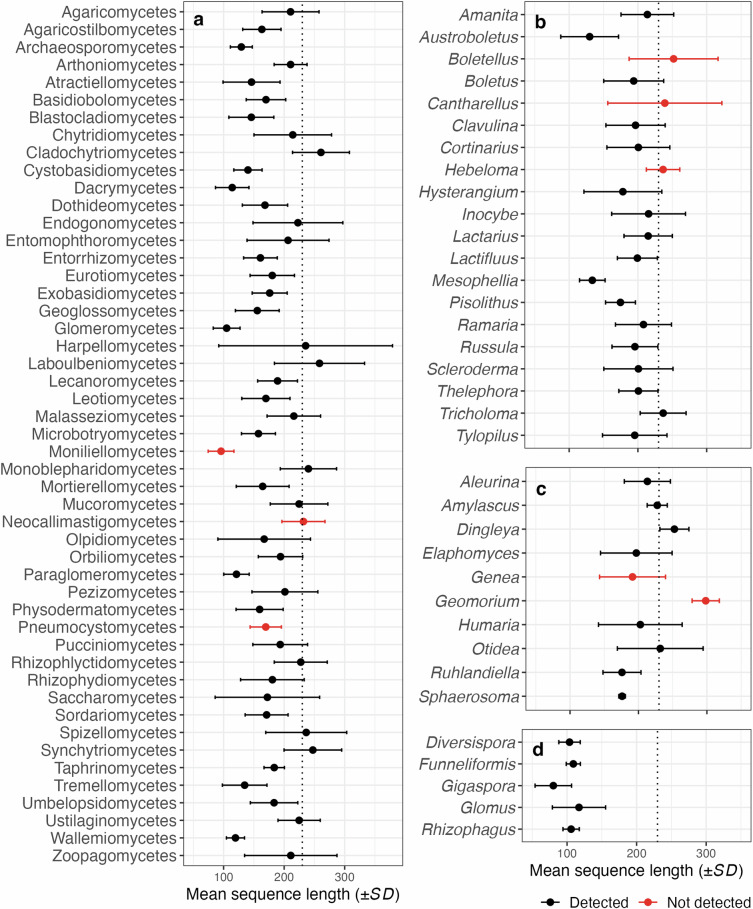
Mean (±SD) ITS1 region length of unique sequences from UNITE + INSD^67^ v10.0 focusing on fungal classes and mycorrhizal fungal genera with described species in Australia based on the Fungi Name Index (https://fungi.biodiversity.org.au). (**a**) Fungal classes, (**b**) ectomycorrhizal (ECM) genera from the class Agaricomycetes, (**c**) ECM genera from phylum Ascomycota, and (**d**) arbuscular mycorrhizal genera from phylum Glomeromycota. Colours illustrate classes and genera that were detected (in black) or not (in red) in our contemporary Australian Microbiome dataset. The detection of classes did not improve when partial ITS1 regions were retained. The dotted line at 230 bp marks the 99th percentile of ITS1 sequence length in our contemporary Australian Microbiome dataset. Classes with a standard deviation extending above this line are likely to contain taxa likely to remain undetected in the dataset.

We processed 300 bp sequences targeting the fungal ITS1, flanked by the highly conserved SSU (18S) and 5.8S rRNA genes. These sequences captured approximately 46 bp of the SSU, with many sequences extending into the 5.8S region, considering that the average length of fungal ITS1 is 177 bp^92^. Removing these conserved regions, which lack species-level resolution, is crucial for enhancing OTU clustering^93^ and taxonomic accuracy^94^. We used ITSxpress^60^ for ITS1 extraction, which is designed to extract ITS regions before denoising. Ideally, forward and reverse reads are merged during ITS extraction, with partial ITS1 sequences discarded to improve ASV calling^60^. Since paired-end reads in the Australian Microbiome fungal dataset cannot be merged (see Bioinformatics workflow), ITS extraction and the discarding of partial ITS1 sequences biases against fungal groups with long ITS1 regions (>230 bp) (Figs. 4–6), likely resulting in false negatives and underestimation of diversity in these groups. To retain partial ITS1 reads, ITS extraction can be performed after quality processing using ITSx^95^. We found that this approach improved the detection of some groups with longer ITS1 regions, such as *Cortinarius* and *Inocybe* (Fig. 5), but coincided with an overall decrease in sequence quality. We acknowledge that discarding partially trimmed ITS1 sequences is conservative and likely results in the exclusion of fungal groups with long ITS1 regions. However, we focused on limiting false positives, which are more deleterious when informing biodiversity platforms^66^ and inferring niche and distribution patterns^65^.

#### Impact of abundance filtering approaches on diversity estimates across fungal phyla

**Fig. 7:**
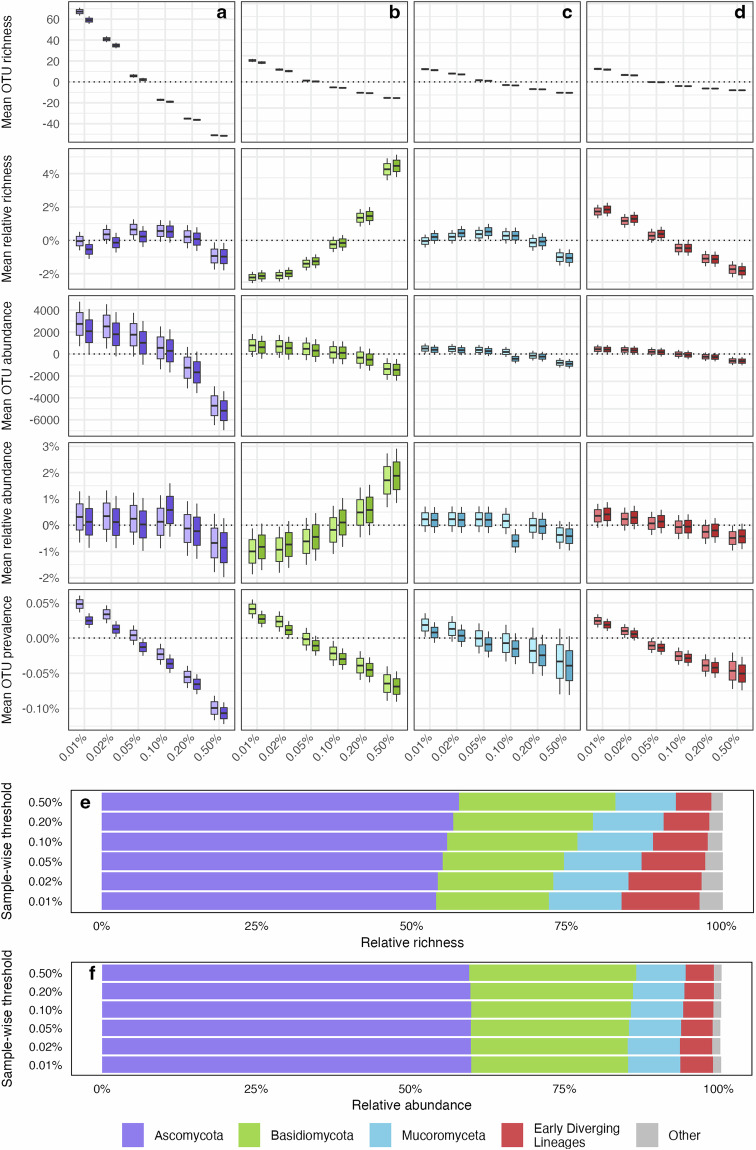
Impact of sample‐wise and library‐wise abundance filtering thresholds on the mean richness (absolute and relative), abundance (absolute and relative), and prevalence of fungal operational taxonomic units (OTUs): (**a**) Phylum Ascomycota, (**b**) phylum Basidiomycota, (**c**) subkingdom Mucoromyceta (comprising phyla Calcarisporiellomycota, Glomeromycota, Mortierellomycota and Mucoromycota), and (**d**) early‐diverging lineages of zoosporic fungi (predominantly phyla Rozellomycota and Chytridiomycota). Sample‐wise thresholds are shown along the x‐axis, and paired boxes represent library‐wise thresholds of 0.5% (on the left) and 1.0% (on the right). Panels (**e**,**f**) demonstrate the impact of sample‐wise filtering on global relative richness and abundance, based on the 0.5% library‐wise abundance filter, which produced a marked effect on richness and a marginal effect on relative sequence abundance at high taxonomic resolution.

To assess the robustness of our filtering approach, we evaluated how sample-wise and library-wise OTU abundance filtering impacted the diversity and prevalence of OTUs across high level groups in our contemporary Australian Microbiome dataset. These groups included phylum Ascomycota, phylum Basidiomycota, subkingdom Mucoromyceta (Calcarisporiellomycota, Glomeromycota, Mortierellomycota and Mucoromycota), and early diverging phyla of unicellular zoosporic fungi^96^ (mostly phyla Rozellomycota and Chytridiomycota) (Fig. 7).

Increasing OTU filtering thresholds generally led to a decrease in mean OTU abundance, richness, and prevalence (Fig. 7). Overall, higher filtering thresholds favoured abundant OTUs in phylum Basidiomycota, while discriminating against OTUs from early diverging phyla. Consequently, our chosen 0.1% sample-wise and 0.5% library-wise cut-offs tended to overestimate the proportional richness of Basidiomycota OTUs to the detriment of early diverging phyla. A similar trend was noted for global OTU richness, however, there was no significant impact on the global relative abundance of sequences (Fig. 7).

#### Impact of sequencing depth on OTU richness

**Fig. 8:**
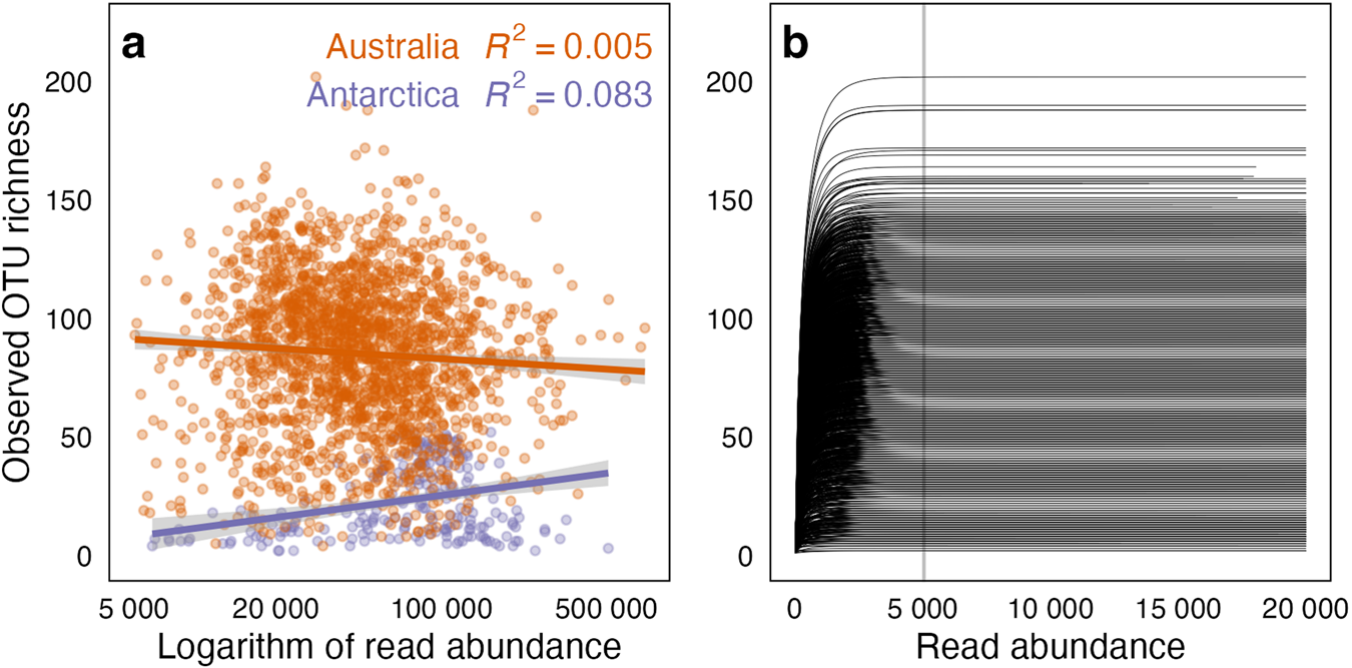
Impact of sequencing depth on operational taxonomic unit (OTU) richness: (**a**) Relationship between sequencing depth and fungal OTU richness in Australian and Antarctic samples; (**b**) rarefaction curves per sample. The vertical grey line represents the minimum sequencing depth with the maximum sequencing depth truncated to 20,000 sequences to improve readability.

We visualised the relationship between sequencing depth and OTU richness, as well as sequencing depth per sample, with rarefaction curves (Fig. 8). Rarefaction curves were in saturation across all samples, and sequencing depth explained <0.1% of the variation in OTU richness within the Australian samples of the contemporary dataset. In contrast, sequencing depth explained 8.5% of the variation in OTU richness in the Antarctic samples (Fig. 8a). These results illustrate that the raw sample-by-OTU matrix does not need rarefaction before conducting diversity analyses on Australian samples, yet diversity analyses specifically focusing on Antarctic samples may benefit from including log-transformed sequencing depth as a covariate in models.

#### Impact of mould abundance on OTU richness

**Fig. 9:**
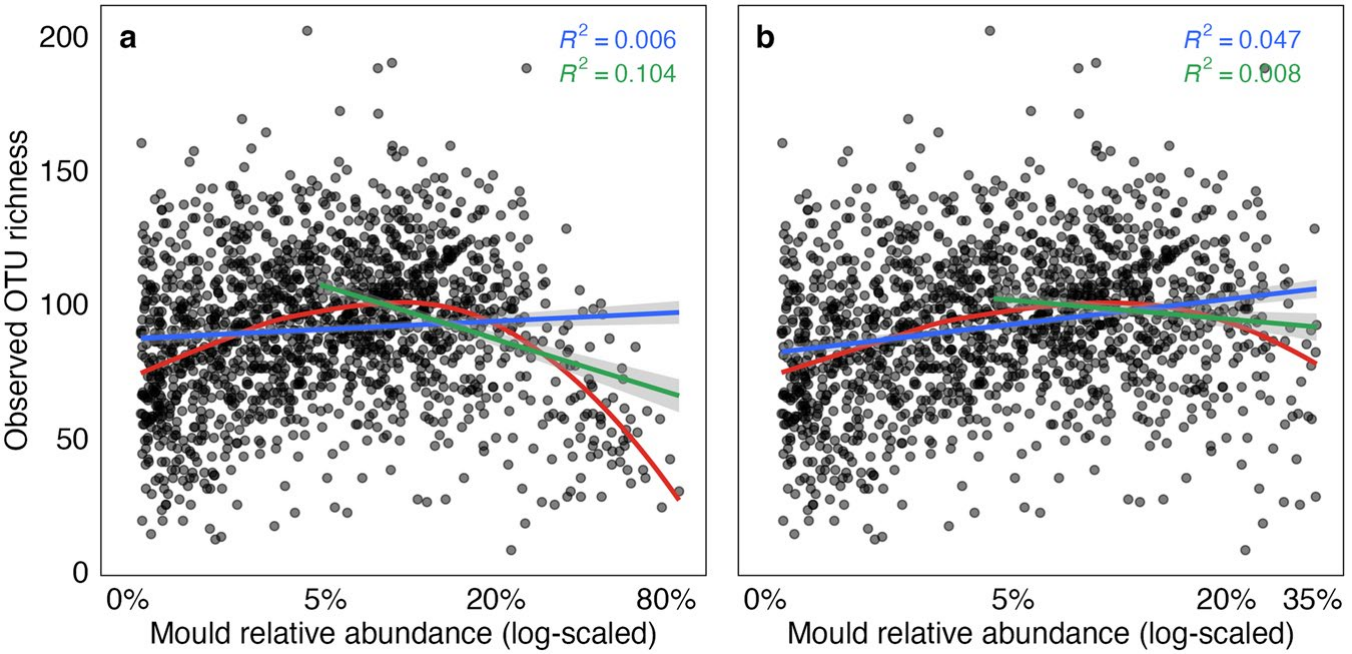
Relationships between mould (Mortierellales, Umbelopsidales and *Trichoderma*) relative abundance and operational taxonomic unit (OTU) richness in our contemporary Australian Microbiome dataset: (**a**) All samples, including those with putative mould contamination, and (**b**) samples without putative mould contamination (i.e. samples with mould relative abundance below the 35% cut‐off). The non‐truncated linear lines (in blue) and Loess curves (in red) represent all data points, while the truncated linear lines (in green) are fitted to data points with mould relative abundance exceeding the median.

Moulds resulting from poor sample preservation can negatively impact sample OTU richness^97^. We tested this by correlating mould relative abundance (i.e. Mucorales, Mortierellales, Umbelopsidales, Aspergillaceae, Trichocomaceae, *Bifiguratus* and *Trichoderma*) with fungal OTU richness in our contemporary dataset. Although mould relative abundance did not affect the overall richness of OTUs, it accounted for 10.1% of the variation in OTU richness in samples with a mould relative abundance greater than the median value (Fig. 9a). We found that the relative abundance of Mortierellales, Umbelopsidales and *Trichoderma* (herein collectively referred to as moulds) had a particularly negative impact on OTU richness, and used their cumulative relative abundance as a proxy for mould contamination. After removing samples with putative mould contamination based on a 35% relative abundance threshold (i.e. mean mould relative abundance plus three standard deviations), the impact of mould relative abundance on OTU richness was drastically reduced (Fig. 9b).

### Spatial validation

#### Tracking ectomycorrhizal distributions as a proxy to index switching

**Fig. 10:**
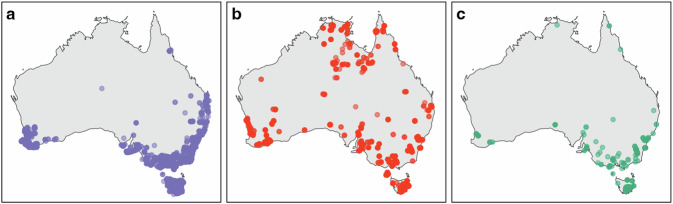
Distribution of *Cortinarius* in Australia: (**a**) Records based on human observations and fungarium specimens^98^, (**b**) amplicon sequence variants (ASVs) from the historical Australian Microbiome dataset on the Atlas of Living Australia^51^, and (**c**) operational taxonomic units (OTUs) from our contemporary dataset.

Considering that no putative ectomycorrhizal host plants exist in Antarctica^53^, nor any human observation or specimens of ectomycorrhizal fungi have been recorded from that region^54^, material sample records of ectomycorrhizal fungi in Antarctica attributed to the Australian Microbiome ITS dataset^51,52^ are likely artefactual. We therefore used these Antarctic samples as ‘environmental controls’ to assess index switching rates based on the detection of ectomycorrhizal OTUs in Antarctica. We further explored biogeographic patterns in the ectomycorrhizal genus *Cortinarius*, a group with a well-described distribution in Australia based on >11,000 human observation and fungarium specimen records^98^. We compared *Cortinarius* distribution in Australia based on observation and specimen records with occurrences from the historical Australian Microbiome dataset on ALA^51^, as well as from our contemporary dataset^52^ (Fig. 10). To make a fair comparison between the contemporary and historical datasets, we limited the latter to topsoil samples and ITS1 sequences, as ALA also houses data from subsoil samples and the ITS2 region.

Our contemporary dataset exhibited 11,379 occurrences of ectomycorrhizal OTUs corresponding to 1,932 OTUs in 81 genera across 1,523 plots (67.4% of all plots), none of which occurred in Antarctica. In contrast, the historical dataset (topsoil ITS1 sequences) detected 24,948 occurrences of ectomycorrhizal ASVs, corresponding to 89 genera across 1,503 plots (87.2% of all plots). The historical dataset contained 818 occurrences of ectomycorrhizal ASVs from 38 genera in Antarctica, including 102 occurrences of *Cortinarius* ASVs. In Australia, *Cortinarius* OTUs in our contemporary dataset followed a similar distribution to human observation and specimen records^98^ (Fig. 10a and c). On the other hand, the historical dataset revealed high *Cortinarius* prevalence in the central and northern regions of Australia where observational records are sparse (Fig. 10a,b). Many of these detections probably include false positives due to index switching in the historical dataset, leading to erroneous distributions in the ALA and GBIF. Such misleading information is likely to be mirrored in other dominant taxa, including important pathogens. Therefore, we advocate for the substitution of the historical Australian Microbiome dataset on the ALA and GBIF with our contemporary dataset.

##### Evaluating the accuracy of taxonomy assignment

We compared our taxonomic assignments with those in the historical Australian Microbiome dataset on Bioplatforms Australia (https://data.bioplatforms.com/bpa/otu), which classified ASVs using the Ribosomal Database Project Classifier and UNITE v9.0. Our contemporary dataset provided taxonomic information for 14,312 OTUs (44.9%) at the genus rank and 2,058 OTUs (6.5%) at the species rank. In contrast, the historical dataset assigned 157,396 (51.3%) ASVs at the genus rank and 35,996 (11.7%) ASVs at the species rank. Each identified species in our dataset corresponded to a single OTU, linking 2,058 unique species to 2,058 unique OTUs. Conversely, the historical dataset linked 35,996 ASVs to 5,358 unique species.

There are currently 8,712 accepted non-lichenised fungal names in Australia, according to the Fungi Names Project (https://fungi.biodiversity.org.au/)—updated using the MycoBank database (https://www.mycobank.org/). We found that only 2,889 of these fungi had sequences in UNITE, suggesting that many species identified in the historical dataset might be linked to sequences from outside Australia.

**Fig. 11:**
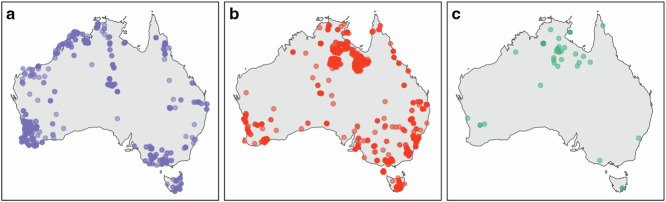
Distribution of Ustilaginomycetes species in Australia: (**a**) Records based on human observation and fungarium specimens^98^, (**b**) amplicon sequence variants from the historical Australian Microbiome dataset on Biopatforms Australia (https://data.bioplatforms.com/bpa/otu), and (**c**) operational taxonomic units from our contemporary dataset.

Our taxonomic assignment approach proved to be comparatively more conservative than the historical dataset. For instance, the historical dataset had 252 ASVs linked to 50 species and 18 genera in the class Ustilaginomycetes (which are mostly plant pathogenic smut fungi), while our contemporary dataset identified 14 species and 10 genera within Ustilaginomycetes. Both datasets extended the known geographic range of many Ustilaginomycetes genera and species, particularly in the savanna region of north-central Australia (Fig. 11). However, many ASVs in the historical dataset had sequences with low similarity to their species annotations. When applying our taxonomic assignment approach to the historical dataset, only 97 of the 252 ASVs remained identified at the class Ustilaginomycetes, with 33 of these receiving new taxonomic annotations. This suggests that our contemporary dataset provides a more conservative but robust species-level annotations and species-distribution estimates.

A recent study highlighted misidentifications of *Amanita* records from the historical Australian Microbiome dataset on the ALA, due to lenient confidence thresholds used during taxonomic assignment^50^. Specifically, 18 unique sequences were misassigned with northern hemisphere taxon names and likely represented native *Amanita* species closely related to those exotic species. To validate the reliability of *Amanita* annotations in our contemporary dataset, we tracked collection locations of sequenced specimens (i.e. source specimens) linked to species hypotheses in UNITE (https://unite.ut.ee/search.php), along with human observation and preserved specimen records in Australia^98^, and compared their distribution with *Amanita* OTUs from our contemporary dataset.

**Fig. 12:**
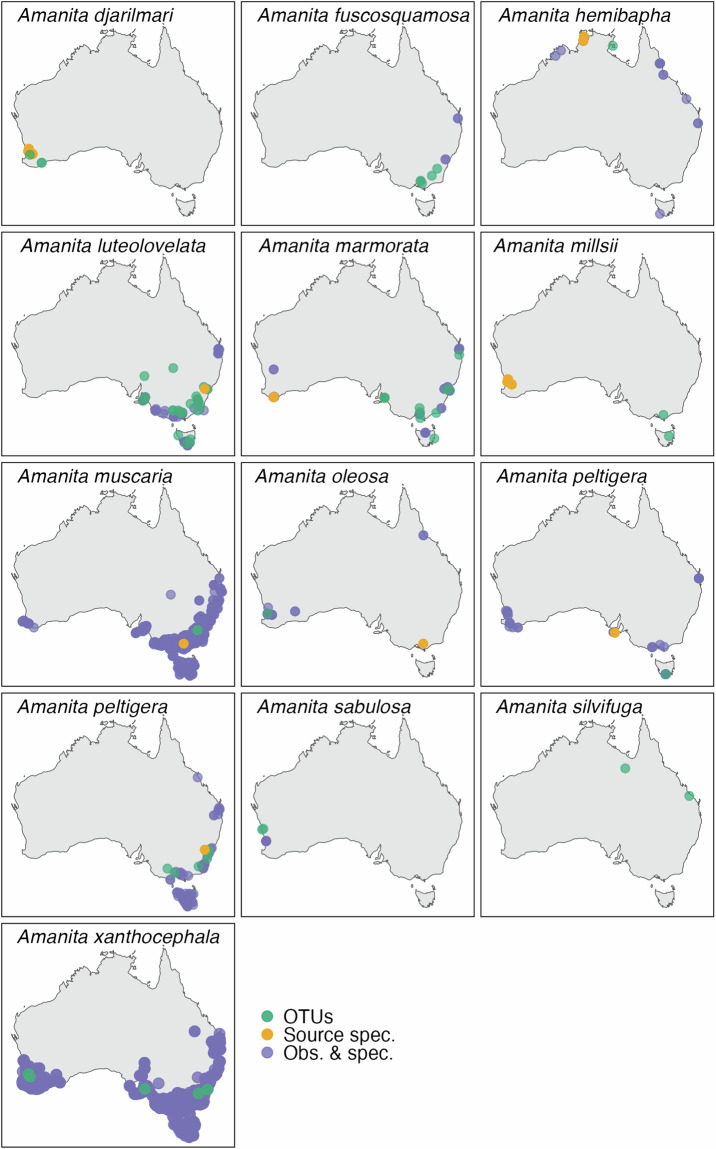
Distribution of *Amanita* in Australia: Operational taxonomic units from our contemporary Australian Microbiome dataset (OTUs; in green), locations of species hypotheses linked to source specimens in UNITE + INSD^67^ (Source spec.; in gold), and human observations and fungarium specimen records^98^ (Obs. & spec.; in purple).

Our contemporary dataset comprised 13 OTUs identified as *Amanita* (Table 3). Among these, 11 specimens had reference sequences originating from Australia. The distribution patterns of these OTUs were typically consistent with source specimens and known locations, yet some exhibited range extensions consistent with known biogeographic patterns in Australia^99^ (Fig. 12). *Amanita muscaria*, known for its global distribution, was also detected, as well as *Amanita silvifuga*, which may be an exotic introduction, though further material is needed for confirmation^50^. These findings suggest that our taxonomic assignment approach using DNAbarcoder^58^ was generally robust and reliably identified *Amanita* fungal OTUs.

**Table 3:** List of *Amanita* species matching to operational taxonomic units (OTUs) in our contemporary Australian Microbiome dataset.

Species detected in our study	Species hypothesis	Region of origin	Similarity score
*Amanita djarilmari*	SH0995436.10FU	Australia	100%
*Amanita fuscosquamosa*	SH0996651.10FU	Australia	99.6%
*Amanita* aff. *hemibapha*	SH0874149.10FU	Australia	100%
*Amanita luteolovelata*	SH0996682.10FU	Australia	100%
*Amanita marmorata*	SH0995436.10FU	Australia	100%
*Amanita millsii*	SH0995436.10FU	Australia	100%
*Amanita muscaria*	SH0963940.10FU	Europe	100%
*Amanita oleosa*	SH0860052.10FU	Australia	99.4%
*Amanita peltigera*	SH0979653.10FU	Australia	100%
*Amanita punctata*	SH0887192.10FU	Australia	100%
*Amanita sabulosa*	SH0993425.10FU	Australia	99.1%
*Amanita silvifuga*	SH0881779.10FU	USA	100%
*Amanita xanthocephala*	SH0858820.10FU	Australia	99.5%

The cut-off for species-level annotations and OTU clustering of *Amanita* sequences was 98.5% similarity in our study.

Note: SH0874149.10FU, which only contains sequences from Australia, was labelled in UNITE as *Amanita hemibapha*, but there are six other UNITE species hypotheses with this name, and the confirmed distribution of this species does not include Australia^109^. Hence, we relabelled SH0874149.10FU as *Amanita* aff. *hemibapha*.

#### Modelling the ecological niches and distribution patterns of mycorrhizal fungi

**Fig. 13:**
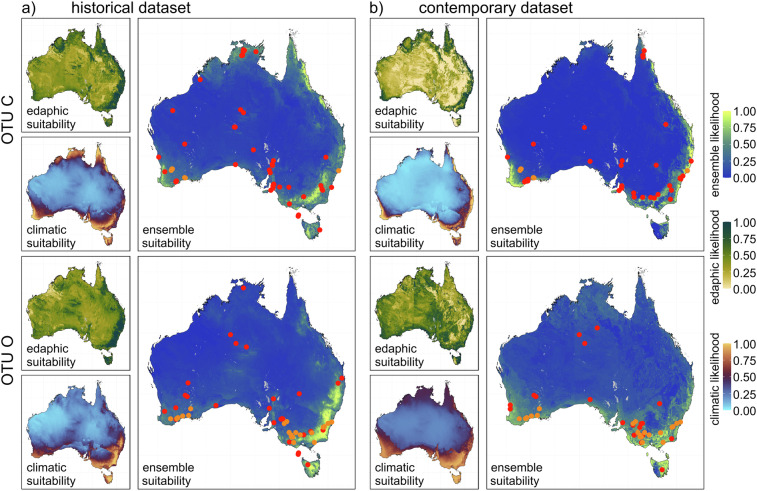
Projected distributions of Ceratobasidiaceae mycorrhizal fungi for operational taxonomic units (OTUs) OTU C (top panel) and OTU O (bottom panel), combining both climatic and edaphic drivers: Projected likelihood of occurrence and proportional contributions to SDMs using the historical Australian Microbiome datasets^25^ (panel a) and the contemporary dataset generated from this study (panel b) as training data. Red points indicate Australian Microbiome data used to train the respective models. Orange points indicate records isolated from orchids^25^.

To assess the performance of our contemporary dataset in species distribution models (SDMs), we replicated SDMs that were previously built for two orchid mycorrhizal fungal OTUs (OTU C and OTU O) from the family Ceratobasidiaceae, based on the historical version of the Australian Microbiome dataset^25^. Presence-background maximum entropy^100,101^ distribution models were created using the R package *dismo*^102^. The SDMs were developed by independently constructing continental-scale climatic and edaphic models, then multiplying these to generate a composite estimate of habitat suitability for each OTU (Fig. 13). We then compared estimations of niche overlap and area of occupancy between the contemporary and historical datasets for each OTU. Detailed methods and results are presented in Supplementary File 1.

The performance of the models for both OTUs appears to be generally robust across datasets, with moderate to high performance scores indicating their reliability in projecting geographical occurrence within the training niche space (Supplementary Table 1). Intraspecific niche overlap between the models was low (OTU C = 0.598; OTU O = 0.269), highlighting marked differences in predictive performance between the historical and contemporary datasets (Fig. 12). Models using our contemporary dataset projected 23,147 km^2^ more suitable area for OTU C and 1,075,343 km^2^ more suitable area for OTU O compared to the historical dataset. Models based on our contemporary dataset appeared more plausible for OTU C, a taxon that is also ectomycorrhizal^103^, therefore with a distribution influenced by host distribution and environmental variables^104^, and less likely to occur across large bioregional gradients. In contrast, models based on our contemporary dataset suggested a more widespread distribution for OTU O than those based on the historical dataset. This modelled distribution matches more closely with known occurrences of OTU O orchid hosts (particularly *Pterostylis spp.*) that are distributed throughout south-west and south-east Australia, including in inland and semi-arid areas^25^. Models based on our contemporary dataset continue to suggest that these fungi have distributions larger than that of their orchid hosts, which is expected as orchid mycorrhizal fungi can have multiple lifestyles^105^, as free-living saprotrophs^106,107^ and ectomycorrhizal fungi with non-orchid plants^103,108^.

## Usage Notes

The contemporary Australian Microbiome dataset generated here is ready-to-use for the detection and ecological modelling of soil fungi in Australia and Antarctica. To account for differences in sequence depth in abundance-based analyses, a normalised OTU-by-sample matrix has been provided. Our dataset can be used without further bioinformatic manipulation or expertise in fungal taxonomy. This dataset is particularly suitable as presence-only data for exploring fungal occurrences and distributions. Our conservative quality filtering approach has likely led to some level of underestimation of fungal diversity, as well as false absences. With this in mind, we recommend that researchers using this dataset for diversity analyses (1) exclude samples with ‘mould contamination’ greater than 35% to account for its negative effect on fungal OTU richness (Fig. 9), (2) note that rarefaction may be required for Antarctic samples, but not for Australian samples (Fig. 8), and (3) keep in mind that the detection rate and diversity of early diverging fungi, as well as fungal groups with long ITS1 regions, are likely to be disproportionately underestimated (Figs. 4–7).

The taxonomic and functional annotations have been rigorously assessed using up-to-date methods for taxonomic assignment and functional reference databases, ensuring robust OTU annotations that overcome underlying quality issues in the raw data. Therefore, we strongly advocate for the integration of our contemporary Australian Microbiome dataset into biodiversity platforms such as the ALA and GBIF, unlocking its immense potential to advance fungal biodiversity and ecology research from local to global scales.

## Supplementary information

Supplementary Information

## Data Availability

The Bash and R scripts and a list of dependencies used to perform the bioinformatics and downstream analyses are available at https://github.com/LukeLikesDirt/AusMycobiome. All primary data products produced in our study are available at figshare^57^.
