# Supplementary material for: A curated soil fungal dataset to advance fungal ecology and conservation research in Australia and Antarctica
Source: Sci Data. 2025 Feb 27;12:353. doi: 10.1038/s41597-025-04598-5 (PMC11868506; doi:10.1038/s41597-025-04598-5)
Supplement: Supplementary file 1 — Supplementary Information [file 41597_2025_4598_MOESM1_ESM.pdf]

Methods

Species distribution modelling

We replicated earlier SDMs for OTU-C and OTU-O (Freestone et al. 2021), using both the observations that they reported for the two taxa, and the refined observation records that we developed here for each taxon. As with previous modelling, we only used the Australian Microbiome Initiative soil sequencing data to train our models and did not use locations of records isolated from orchid symbioses.

As with earlier SDMs of soil microbes, we used MaxEnt version 3.3.3a (Phillips et al. 2006; Phillips and Dudik 2008) implemented in the R package 'dismo' (Hijmans et al. 2017) to produce presence-background models of distribution. The number of records for each OTU differed according to the two refinements of the Australian Microbiome Initiative data (Table S1). However, in each model we removed duplicate records within single grid cells were removed (Elith et al. 2011; Newbold et al. 2010). To account for stochasticity of the random background, we constructed five independent background (pseudoabsence) data sets (Elliott et al. 2024), each 100 times the number of unique occurrence locations of each OTU (i.e. the number of occupied grid cells with all duplicate records removed). We optimised the modelling parameters using the *ENMevaluate* algorithm in the R package 'ENMeval' (Kass et al. 2021) by iteratively tuning both the regularization multiplier (0.0-2.0 at intervals of 0.2) and the feature classes (linear and linear-quadratic; Elith et al. (2011); Merow et al. (2013). For each combination of parameter settings, we evaluated the model against five unique background draws, and assessed the model performance using the ensemble average. The combination that consistently produced the lowest delta AICc was used to define the MaxEnt parameter settings (Table S1). For each independent replicate, we to developed logistic likelihoods of habitat suitability, ranging from zero at the lowest likelihood of presence to one at the strongest prediction for presence (Phillips et al. 2006).

The MaxEnt distribution models were constructed in two phases. In the first phase, we replicated earlier climatically-informed models (Freestone et al. 2021), using Bioclim parameters (Hijmans et al. 2005; Xu and Hutchinson 2011), calculated at 30 arc-second resolution (~1 km^2). The climatically-informed models were constructed using mean temperatures of the wettest (Bio08) and driest quarters (Bio09), the mean temperatures of the warmest (Bio10) and coolest quarters (Bio11), the mean soil moisture indices of the wettest (Bio32) and driest quarters (Bio33), and the mean soil moisture indices of the warmest (Bio34) and coolest quarters (Bio35). The second phase of SDMs were developed using geomorphological and edaphic data that have previously proven useful in modelling

the distributions of short-range endemic plants (Lewandrowski et al. 2024; Tomlinson et al. 2019). These data represent elevation, aspect, and slope (Gallant and Austin 2012a, b; Gallant et al. 2011), clay, sand, and silt percentage at 15 cm depth (Viscarra Rossel et al. 2014a, c, d), pH (Viscarra Rossel et al. 2014b) and soil bulk density and depth (interpolated from national soil data provided by the Australian Collaborative Land Evaluation Program ACLEP, endorsed through the National Committee on Soil and Terrain NCST (www.clw.csiro.au/aclep). As with earlier efforts to refine the bioclimatic correlates of distribution, we constructed a pilot model using all the edaphic and geomorphological correlates, and then rendered the most parsimonious model using the *dredge* and *model.avg* functions in the 'MuMIn' package in R (Barton 2013). The final model was constructed using elevation and slope, clay, sand, and silt composition, pH and soil bulk density. In the final phase, continental-scale projections of the climatic and edaphic models were multiplied to generate a composite estimate of habitat suitability for each microbial taxon.

We evaluated model performance by calculating the area under the area under the threshold-independent receiver-operating characteristic (ROC) curve (AUC), using values >0.9 to indicate well-validated models (Swets 1988). We also calculated the True Skill Score (TSS) as a test of model robustness (Allouche et al. 2006; Williams et al. 2009) using the *evalSDM* function in the 'mecofun' v0.1.1 package (Zurell 2020). Models with TSS < 0.4 were identified as poor, while models with TSS > 0.6 were identified as performing well (Beauregard and de Blois 2014). We calculated a Boyce index of correlation between presence and suitability (Boyce et al. 2002) using the *ecospat.boyce* function in the 'ecospat' package (Di Cola et al. 2017) These performance metrics were calculated over 100-iteration bootstraps using 10% test presence, which reserves 10% of the known occurrence locations for testing the resulting models (Phillips et al. 2006; Phillips and Dudik 2008). Finally, a weighted ensemble mean of habitat suitability for both climatically-informed and edaphically-informed models was calculated, where models with higher TSS were weighted more heavily in the average than models with lower correlation scores (Elliott et al. 2024).

To assess the difference in potential distributions caused by changes in the identification of known locations between the Australian Microbiome Initiative data used by Freestone et al. (2021), and the location data developed here, we calculated niche overlap within taxa between the two model versions. Niche overlap was estimated by calculating Schoener's D from the combined habitat suitability projection (i.e. incorporating both climatic and edaphic models) using the *calc.niche.overlap* function in the 'ENMeval' package (Kass et al. 2021). We also estimated the difference in potential area of occupancy between the two iterations of the SDM. To do this, we converted the ensemble

model projections to binary estimates using the TSS threshold as our limit to the habitable niche, and calculated the area of habitat above this threshold using the *cellSize* function of the 'terra' R package (Hijmans 2023).

Results

Species distribution modelling

The climate-only projections using the location data reported by Freestone et al. (2021) remain highly consistent, with moderate likelihood of occurrence of OTU C around much of the periphery of continental Australia and OTU O modelled to occur across the majority of southern, eastern and northern Australia (Figure 1). The addition of an edaphic filter, however, substantially altered the areas of projected habitat suitability, restricting OTU C to the southwest of Western Australia, and eastern coastal regions particularly coastal areas of north-east Queensland (east of the Great Dividing Range), and mountainous areas of south-eastern Victoria and New South Wales and Tasmania (Figure 1). Primarily, the edaphic filter restricted OTU C to relatively shallow slopes with low bulk density (Figure S1 and S2). The addition of an edaphic filter constrained the distribution of OTU O even more severely, modelling the species to occur only in high altitude regions of Victoria, New South Wales and Tasmania (Figure 1), in alkaline, low elevation soils (Figure S1 and S2).

By comparison, when modelled using the newer occurrence data, the distribution of OTU C was most strongly correlated with relatively low moisture in the coldest quarter, and relatively high moisture in the wettest quarter and steeper, sandy soils (Figure S1 and S2). The resulting combined distribution showed high likelihoods of occurrence in south-west Australia, south-eastern coastal and mountainous regions, and up the eastern coast to monsoonal north Queensland (Figure 1). The highest likelihoods of occurrence (ranging from 88-97 %) were projected at locations along the southeastern coastline, from the tip of the Fleurieu Peninsula in South Australia to Cape York. A total of 73% of presence locations occurred within the modelled binary area of occupancy. The distribution of OTU O was most strongly correlated with relatively low moisture in the coldest quarter and low bulk density, sandy soils (Figure S1 and S2). The resulting combined distribution showed much more diverse distribution of likely occurrence across most of southern Australia, specifically the southwest and southeastern parts of the continent, and eastern Tasmania (Figure 1). The highest likelihood of occurrence (75 %) was projected at an occurrence location in southeastern Victoria and Tasmania. A total of 86% of presence locations occurred within the modelled binary area of occupancy.

The contemporary (2024 data) models for both OTUs appear to be generally robust; climate-only models returned AUC > 0.7 (OTU C = 0.852, OTU O = 0.764), and TSS of ~0.6 (OTU C = 0.706, OTU O = 0.625), while edaphic models returned AUC > 0.7 (OTU C = 0.881, OTU O = 0.796), and TSS of ~0.6 (OTU C = 0.732, OTU O = 0.665). Intraspecific niche overlap between the models trained with the Freestone et al. (2021) occurrence data and the contemporary occurrence data was low (OTU C = 0.598; OTU O = 0.269), projecting 23,147 km^2 more suitable area for OTU C, and 1,075,343 km^2 more suitable area for OTU O.

Table S1: Number of training points and optimised parameters contributing to each iteration of the MaxEnt models for each mycorrhizal fungus OTU. N represents the total number of records, while (n) represent the unique training records after duplicates have been removed. “L” indicates a linear response function, and “LQ” indicates a linear-quadratic response function.

		Taxon	2021 training data						2024 training data					
			**N (n)**	**curve**	**multiplier**	**AUC**	**TSS**	**Boyce**	**N (n)**	**curve**	**multiplier**	**AUC**	**TSS**	**Boyce**
climatic models	OTU C	Replicate 1				0.805	0.615	0.824				0.854	0.711	0.547
		Replicate 2				0.794	0.602	0.791				0.864	0.713	0.579
		Replicate 3				0.797	0.607	0.824				0.853	0.717	0.664
		Replicate 4				0.813	0.638	0.787				0.839	0.689	0.630
		Replicate 5				0.774	0.579	0.812				0.851	0.700	0.656
			**54 (49)**	**L**	**0.2**	**0.796**	**0.608**	**0.808**	**48 (35)**	**L**	**0.01**	**0.852**	**0.706**	**0.615**
	OTU O	Replicate 1				0.891	0.770	0.726				0.770	0.623	0.695
		Replicate 2				0.912	0.804	0.693				0.757	0.625	0.594
		Replicate 3				0.930	0.839	0.755				0.784	0.656	0.713
		Replicate 4				0.906	0.800	0.730				0.758	0.610	0.700
		Replicate 5				0.908	0.795	0.765				0.750	0.612	0.634
			**46 (39)**	**LQ**	**1.2**	**0.909**	**0.801**	**0.734**	**21 (20)**	**L**	**1.26**	**0.764**	**0.625**	**0.667**
edaphic models	OTU C	Replicate 1				0.737	0.557	0.711				0.864	0.700	0.821
		Replicate 2				0.764	0.592	0.700				0.877	0.725	0.870
		Replicate 3				0.750	0.568	0.687				0.900	0.760	0.866
		Replicate 4				0.758	0.564	0.734				0.890	0.749	0.882
		Replicate 5				0.758	0.582	0.701				0.876	0.724	0.884
			**54 (49)**	**L**	**0.8**	**0.753**	**0.572**	**0.707**	**48 (35)**	**LQ**	**1.2**	**0.881**	**0.732**	**0.865**
	OTU O	Replicate 1				0.835	0.676	0.625				0.799	0.662	0.713
		Replicate 2				0.848	0.699	0.623				0.794	0.668	0.523
		Replicate 3				0.836	0.662	0.626				0.803	0.676	0.471
		Replicate 4				0.847	0.691	0.647				0.801	0.667	0.591
		Replicate 5				0.823	0.656	0.670				0.783	0.649	0.548
			**46 (39)**	**L**	**0.2**	**0.838**	**0.677**	**0.638**	**21 (20)**	**LQ**	**1.0**	**0.796**	**0.665**	**0.569**

Commented [ST1]: Same as with thee previous: this is better in the main MS. I'd drop it here

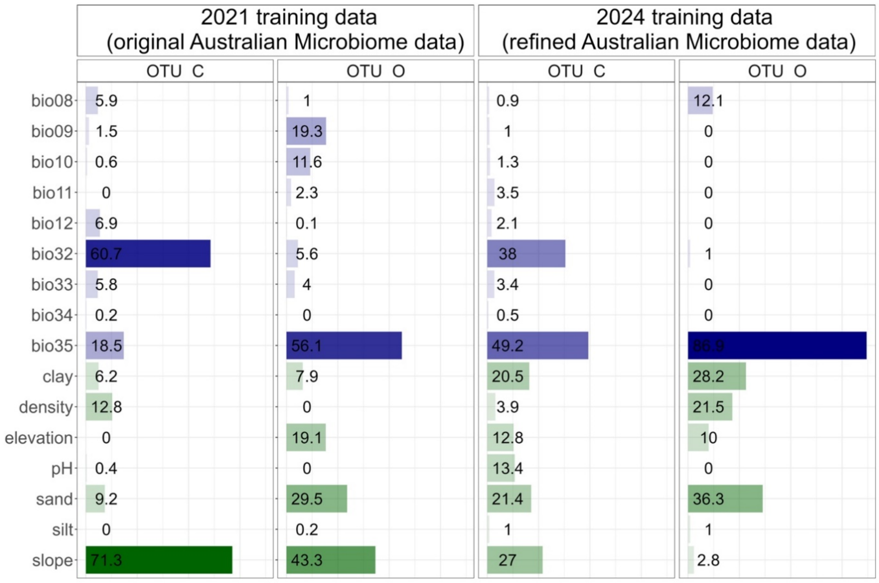

Figure S1. Variable importance climatic and edaphic drivers of distributions of mycorrhizal fungi OTU C and OTU O.

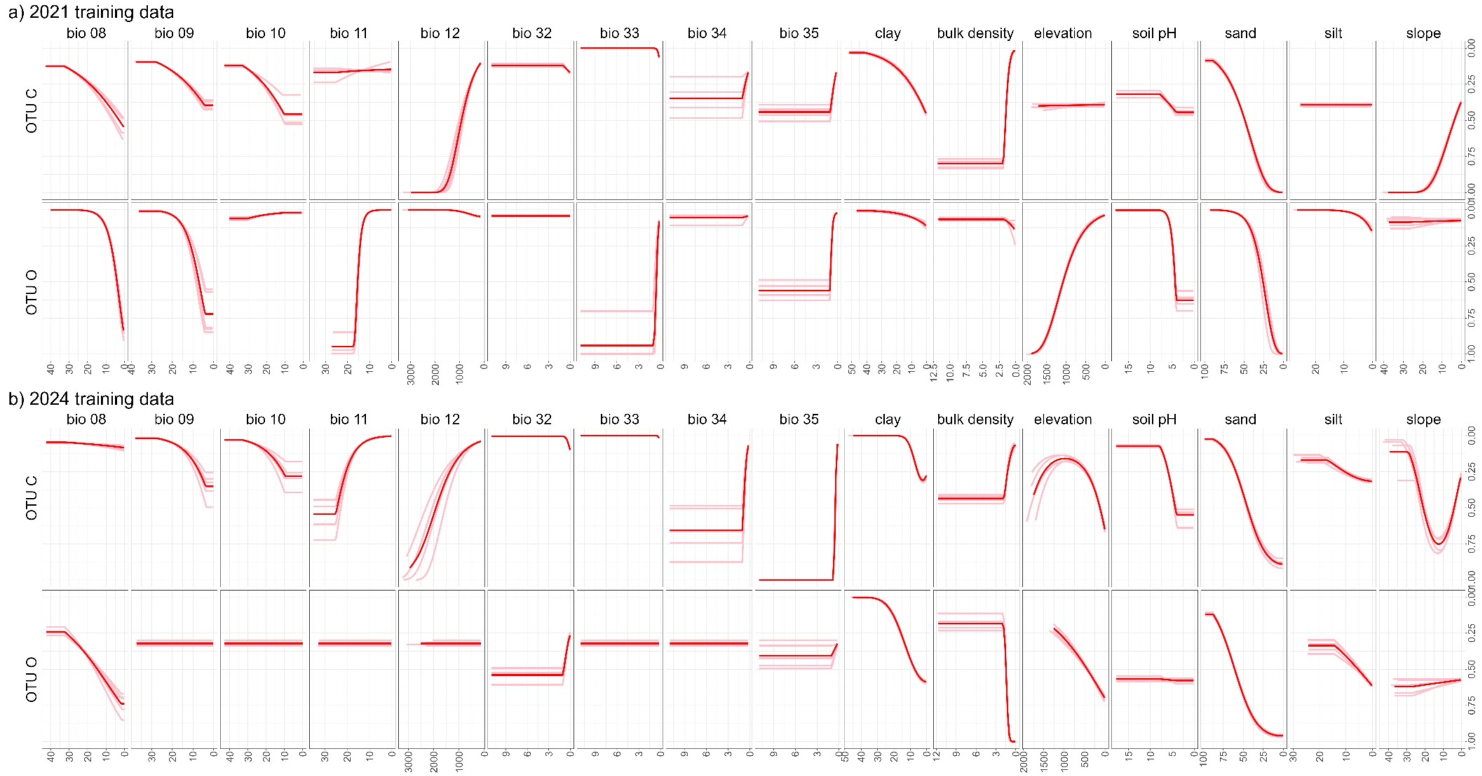

Figure S2: Response plots showing patterns of effect of both climatic and edaphic drivers contributing to **a)** the models informed by location data reported by Freestone et al. (2021), and **b)** drivers of the models informed by the refined occurrence data reported here.

References

Allouche, O., Tsoar, A., Kadmon, R., 2006. Assessing the accuracy of species distribution models: prevalence, kappa and the true skill statistic (TSS). Journal of Applied Ecology 43, 1223-1232.

Barton, K., 2013. MuMIn: Multi-model inference version. R package version 1.9.13.

Beauregard, F., de Blois, S., 2014. Beyond a climate-centric view of plant distribution: edaphic variables add value to distribution models. PLoS ONE 9, e92642.

Boyce, M.S., Vernier, P.R., S.E., N., Schmiegelow, F.K.A., 2002. Evaluating resource selection functions. Ecological Modelling 157, 281-300.

Brown, S.C., Wigley, T.M., Otto-Bliesner, B.L., Fordham, D.A., 2020. StableClim, continuous projections of climate stability from 21000 BP to 2100 CE at multiple spatial scales. Scientific Data 7, 1-13.

Di Cola, V., Broennimann, O., Petitpierre, B., Breiner, F.T., D'amen, M., Randin, C., Engler, R., Pottier, J., Pio, D., Dubuis, A., Pellissier, L., 2017. ecospat: an R package to support spatial analyses and modeling of species niches and distributions. Ecography 40, 774-787.

Elith, J., Phillips, S.J., Hastie, T., Dudík, M., Chee, Y.E., Yates, C.J., 2011. A statistical explanation of MaxEnt for ecologists. Diversity and Distributions 17, 43-57.

Elliott, C.P., Tomlinson, S., Lewandrowski, W., Miller, B.P., 2024. Species distribution and habitat attributes guide translocation planning of a threatened short-range endemic plant. Global Ecology and Conservation p.e02915.

Freestone, M.W., Swarts, N.D., Reiter, N., Tomlinson, S., Sussmilch, F.C., Wright, M.M., Holmes, G.D., Phillips, R.D., Linde, C.C., 2021. Continental-scale distribution and diversity of *Ceratobasidium* orchid mycorrhizal fungi in Australia. Annals of Botany 128, 329-343.

Gallant, J.C., Austin, J., 2012a. Aspect derived from 1" SRTM DEM-S v6., In CSIRO Data Collection. http://doi.org/10.4225/08/56D778315A62B

Gallant, J.C., Austin, J., 2012b. Slope derived from 1" SRTM DEM-S. v4., In CSIRO Data Collection. http://doi.org/10.4225/08/5689DA774564A

Gallant, J.C., Dowling, T.I., Read, A.M., Wilson, N., Tickle, P., Inskeep, C., 2011. 1 second SRTM derived digital elevation models user guide. Geoscience Australia, Canberra, Australia.

Hijmans, R.J., 2023. terra: Spatial Data Analysis., The Comprehensive R Archive Network.

Hijmans, R.J., Cameron, S.E., Parra, J.L., Jones, P.G., Jarvis, A., 2005. Very high resolution interpolated climate surfaces for global land areas. . International Journal of Climatology 25, 1965-1978.

Hijmans, R.J., Phillips, S., Leathwick, J., Elith, J., Hijmans, M.R.J., 2017. Package 'dismo'. Circles 9, 1-68.

Kass, J.M., Muscarella, R., Galante, P.J., Bohl, C.L., Pinilla-Buitrago, G.E., Boria, R.A., Soley-Guardia, M., Anderson, R.P., 2021. ENMeval 2.0: Redesigned for customizable and reproducible modeling of species' niches and distributions. Methods in Ecology and Evolution 12, 1602-1608.

Lewandrowski, W., Tudor, E.P., Ajduk, H., Tomlinson, S., Stevens, J.C., 2024. Spatiotemporal variation in ecophysiological traits align with high resolution niche modelling in the short-range banded ironstone endemic *Aluta quadrata*. Conservation Physiology IN PRESS.

Merow, C., Smith, M.J., Silander, J.A.J., 2013. A practical guide to MaxEnt for modeling species' distributions: what it does, and why inputs and settings matter. Ecography 36, 1058-1069.

Newbold, T., Reader, T., El-Gabbas, A., Berg, W., Shohdi, W.M., Zalat, S., El Din, S.B., Gilbert, F., 2010. Testing the accuracy of species distribution models using species records from a new field survey. Oikos 119, 1326-1334.

Phillips, S.J., Anderson, R.P., Schapire, R.E., 2006. Maximum entropy modeling of species geographic distributions. Ecological Modelling 190, 231-259.

Phillips, S.J., Dudik, M., 2008. Modeling of species distributions with Maxent: new extensions and a comprehensive evaluation. Ecography 31, 161-175.

Swets, J., 1988. Measuring the accuracy of diagnostic systems. Science 240, 1285-1293.

Tomlinson, S., Lewandrowski, W., Elliott, C.P., Miller, B.P., Turner, S.R., 2019. High resolution distribution modelling of a threatened short-range endemic plant informed by edaphic factors. Ecology and Evolution 10, 763-777.

Viscarra Rossel, R., Chen, C., Grundy, M., Searle, R., Clifford, D., Odgers, N., Holmes, K., Griffin, T., Liddicoat, C., Kidd, D., 2014a. Soil and Landscape Grid National Soil Attribute Maps - Clay (3" resolution) - Release 1. v4., In CSIRO Data Collection. http://doi.org/10.4225/08/546EEE35164BF.
Viscarra Rossel, R., Chen, C., Grundy, M., Searle, R., Clifford, D., Odgers, N., Holmes, K., Griffin, T., Liddicoat, C., Kidd, D., 2014b. Soil and Landscape Grid National Soil Attribute Maps - pH - CaCl2 (3" resolution) - Release 1. v3., In CSIRO Data Collection. https://doi.org/10.4225/08/546F17EC6AB6E.
Viscarra Rossel, R., Chen, C., Grundy, M., Searle, R., Clifford, D., Odgers, N., Holmes, K., Griffin, T., Liddicoat, C., Kidd, D., 2014c. Soil and Landscape Grid National Soil Attribute Maps - Sand (3" resolution) - Release 1. v4., In CSIRO Data Collection. http://doi.org/10.4225/08/546F29646877E
Viscarra Rossel, R., Chen, C., Grundy, M., Searle, R., Clifford, D., Odgers, N., Holmes, K., Griffin, T., Liddicoat, C., Kidd, D., 2014d. Soil and Landscape Grid National Soil Attribute Maps - Silt (3" resolution) - Release 1. v4., In CSIRO Data Collection. http://doi.org/10.4225/08/546F48D6A6D48
Williams, J.N., Seo, C., Thorne, J., Nelson, J.K., Erwin, S., O'Brien, J.M., Schwartz, M.W., 2009. Using species distribution models to predict new occurrences for rare plants. Diversity and Distributions 15, 565-576.
Xu, T., Hutchinson, M., 2011. ANUCLIM version 6.1 user guide. The Australian National University, Canberra.
Zurell, D., 2020. mecofun: Useful functions for macroecology and species distirbution modelling. University of Potsdam, Potsdam, Germany.
